# Spatial Analysis of HIV Infection and Associated Risk Factors in Botswana

**DOI:** 10.3390/ijerph18073424

**Published:** 2021-03-25

**Authors:** Malebogo Solomon, Luis Furuya-Kanamori, Kinley Wangdi

**Affiliations:** Department of Global Health, Research School of Population Health, Australian National University, Canberra, ACT 2601, Australia; Luis.Furuya-Kanamori@anu.edu.au (L.F.-K.); kinley.wangdi@anu.edu.au (K.W.)

**Keywords:** Botswana, HIV, spatial analysis, clusters, risk factors

## Abstract

Botswana has the third highest human immunodeficiency virus (HIV) prevalence globally, and the severity of the epidemic within the country varies considerably between the districts. This study aimed to identify clusters of HIV and associated factors among adults in Botswana. Data from the Botswana Acquired Immunodeficiency Syndrome (AIDS) Impact Survey IV (BIAS IV), a nationally representative household-based survey, were used for this study. Multivariable logistic regression and Kulldorf’s scan statistics were used to identify the risk factors and HIV clusters. Socio-demographic characteristics were compared within and outside the clusters. HIV prevalence among the study participants was 25.1% (95% CI 23.3–26.4). HIV infection was significantly higher among the female gender, those older than 24 years and those reporting the use of condoms, while tertiary education had a protective effect. Two significant HIV clusters were identified, one located between Selibe-Phikwe and Francistown and another in the Central Mahalapye district. Clusters had higher levels of unemployment, less people with tertiary education and more people residing in rural areas compared to regions outside the clusters. Our study identified high-risk populations and regions with a high burden of HIV infection in Botswana. This calls for focused innovative and cost-effective HIV interventions on these vulnerable populations and regions to curb the HIV epidemic in Botswana.

## 1. Introduction

Although the first human immunodeficiency virus (HIV) case was identified almost four decades ago, it remains a significant public health issue, affecting approximately 36.7 million people globally [1]. Sub-Saharan Africa (SSA) bears the highest burden of HIV, accounting for approximately 70% of the total number of people living with HIV globally [2,3]. Despite the introduction of antiretroviral therapy (ART), HIV/Acquired Immune Deficiency Syndrome (AIDS) remains one of the leading causes of death in SSA [2]. There is increasing evidence suggesting that HIV epidemics are heterogenous and that HIV transmission is mostly concentrated within clustered micro-epidemics of varying geographical scales. To adequately mitigate the HIV/AIDS epidemic, it is imperative to employ geo-analytical methods to locate these clusters and understand the underlying determinants to optimise HIV prevention and treatment interventions currently in place [4,5].

Botswana, like most SSA countries, is battling a high burden of HIV. With a national prevalence rate of 23.7%, it has the third highest number of cases globally, with 13,800 newly diagnosed cases reported in 2017 [6]. The country introduced several interventions to fight the epidemic, including, but not limited to, ART, distribution of free condoms and safe male circumcision (SMC) [7]. The introduction of ART in 2002 increased the country’s life expectancy from 49 years in 2000 to 64 years in 2013 [8]. SMC was introduced to reduce HIV incidence and prevalence and by 2016, 42.7% of the targeted population had been circumcised [9]. These interventions contributed to the decline of the country’s prevalence rate from 26% in 2007 to 23.7% in 2017 [6,10]. However, varying prevalence rates have been reported across the districts over the past years. Some districts such as Chobe have experienced a decline in prevalence rates (from 29.4% in 2004 to 17.7% in 2013), while other districts such as Barolong and Kweneng East have shown an increasing trend (from 14.4% and 15.2% in 2004 to 20.3% and 21.5% in 2013, respectively) [11]. This sub-national variation in prevalence exists not only across districts but also between genders and age groups, indicating that Botswana’s current HIV prevalence is highly heterogenous [11,12]. The persistent high and varying geographic prevalence rates indicate that the one-size-fits-all approach to HIV prevention that the country has adopted is not effective, hence the need for a more effective geographical prioritization approach as recommended by the Joint United Nations Programme on HIV/AIDS [13].

To design effective HIV interventions requires an understanding of the spatial distribution of HIV prevalence and risk factors associated with HIV transmission. Previous studies have demonstrated that demographic and socio-economic factors are important predictors of HIV transmission in Botswana [12,14,15]. However, because of difficulties in obtaining geolocated HIV data, studies conducted in Botswana have mostly been conducted in large geographic units such as at the district level [15]. This coarse scale has necessitated assumptions about the characteristics, size and location of the study population, in the process masking important sub-area variation [16]. For instance, studies by Chomoyi and Musenge in Uganda [17] and Tanser et al. in KwaZulu Natal Province South Africa [16] were able to identify clusters, indicating the presence of micro-epidemics in otherwise generalised epidemics. These would not have been found without the use of a geo-spatial analytical approach; these studies have been instrumental in the implementation of geographically targeted, and comparatively cost-effective HIV interventions [18]. Botswana would greatly benefit from such focused interventions, especially considering that currently US$188 per capita, 44% of the country’s total health expenditure, is being spent on HIV and AIDS [19]. Therefore, we used the Botswana AIDS Impact Survey (BIAS) IV data and incorporated spatial analysis methods to investigate spatial heterogeneity of HIV prevalence in Botswana and identify risk factors associated with HIV infection. 

## 2. Materials and Methods

### 2.1. Study Area

The study was carried out in Botswana, a land-locked country in southern Africa that shares borders with South Africa, Zimbabwe, Zambia and Namibia and covers an area of about 582,000 km^2^. Botswana is divided into 10 administrative districts, and further divided into 28 sub-districts, also referred to as census districts (as of 2011). The Population and Housing Census of 2011 estimated Botswana’s population to be 2,024,904.

### 2.2. Study Data

A cross sectional study was conducted on a national population-based household survey, the Botswana AIDS Impact Survey IV (BIAS IV), which was carried out between January and April 2013. The survey is the latest of a series conducted by Botswana Central Statistics with the mandate to provide current HIV incidence and prevalence estimates as well as HIV knowledge in Botswana. The sample frame for the survey was provided by the 2011 Botswana Population and Housing Census, and a stratified two-stage sampling design was employed. The first stage involved random selection of primary sampling units, which were stratified by district and place of residence (urban/rural). In the second stage, an estimated 25 households per enumeration area were randomly selected, resulting in [20]. 

In the survey, a total of 9807 participants from the selected households aged 10–64 years were eligible to complete the individual questionnaire but only 8231 (83.9%) completed the survey. Demographic, sexual history and HIV/AIDS knowledge details of study participants were captured on a standardized questionnaire loaded on an Open Data Kit (ODK). Study participants who consented gave a dried blood spot sample which was later tested for HIV antibodies using the commercial HIV testing kits Vironostika and Murex. 

The current study focused on adults ≥15 years of age and participants with missing data on covariates, HIV status and geolocation were excluded, giving a final study sample of 4708 participants.

### 2.3. Measures

The primary outcome variable for this study was HIV status, which was measured for all participants and divided into two categories—positive and negative. HIV predictor variables included socio-demographic factors—gender (male, female), age category (15–24, 25–34, 35–44, 45–54 and 55+ years), residence (urban, rural), marital status (never married, married/ever married), highest education level attained (none, primary, secondary, tertiary), religion (Christian, other) and employment (yes, no). HIV-related risk factors included alcohol use (yes, no) and condom use (yes, no). Data on these factors were considered based on the Joint United Nations Programme on HIV and AIDS (UNAIDS) guidelines for second-generation surveillance [21].

### 2.4. Statistical Analysis

Stata software version 14.1 (Stata Corp, College Station, TX, USA) was used to perform all statistical analyses. Sample weights accounting for complex survey design were incorporated in all calculations using the ‘svy’ module in Stata.

Standard descriptive statistics such as median and interquartile range for continuous variables and frequencies and proportions for categorical data were calculated to characterise HIV prevalence by socio-demographic and HIV-related risk factors. Univariate analysis using tabulation, a chi-square test and logistic regression were used to assess the association between covariates and HIV status. Multivariable logistic analysis was performed on covariates with *p* value < 0.1 in the univariate analysis. Unadjusted odds ratios (ORs) and adjusted odds ratios (AORs) and their 95% confidence intervals (CI) with a *p*-value < 0.05 were considered statistically significant results.

### 2.5. Spatial Analyses

The spatial units were the 28 census districts of Botswana as delineated by the 2011 Population and Housing Census. Census districts comprise the second administrative level in Botswana. Open data Kit with geographic information system (GIS) and global positioning system (GPS) technologies were used to collect the geographic coordinates (longitude and latitude) of the EAs. All study participants were geolocated as per their households. First, we calculated the crude estimation of HIV prevalence for all the 28 census districts and used the results to develop a choropleth map.

Second, spatial cluster analysis was done with Kulldorff’s scan statistic, using the SaTScan™ software [22]. The spatial scan statistic identifies statistically significant clusters using scanning windows (circular or elliptical) of varying sizes. These windows move around the study area to identify HIV-positive individuals within a window that is more than what is expected by chance. The relative risk (RR) of positive cases for each window is calculated using the observed number of positive and negative individuals within the windows [23,24]. The highest log-likelihood ratio (LLR) was calculated for each cluster to determine the most likely cluster. A Bernoulli Probability Model was used for this study because of the binomial outcome of interest (HIV-positive and -negative). We ran multiple analyses by setting the maximum spatial cluster size of 50%, 25%, 20% and 15% of the population at risk of HIV infection. The best cluster size was obtained at the maximum spatial cluster size of 25%. The statistically significant clusters with the radius of the scanning window, the number of observed and expected positives within the circle, RR, LLR and *p* value were saved for analysis and mapping. Socio-demographic characteristics of participants living within the cluster were compared against those living outside the clusters using a Chi-square test. All analyses were run using Monte Carlo replications of 999 for defining clusters and *p* < 0.05 was considered significant. The significant clusters obtained by SaTScan™ were mapped using ArcMap 10.5.1 (ESRI, Redlands, CA, USA). 

## 3. Results

### 3.1. Socio-Demographic and Behavioural Characteristics of Participants

Overall, 4708 participants were included in the analysis. Females made up 56.4% of the study participants, and the majority of participants (51.8%) were educated up to secondary level. Over half had never been married (56.1%) and 64.6% resided in urban areas (cities, towns and urban villages) (Table 1). The prevalence of HIV among the study participants was 25.1% (95% CI 23.3–26.4). 

In the univariate analysis, gender, age, marital status, education level, employment and condom use were significantly associated with HIV status, while residence, alcohol use and religion were not statistically significant. After adjusting for all other variables included in the multivariable logistic regression model, independent predictors of increased odds of HIV infection were being female (adjusted odds ratio (AOR) = 1.42, 95% CI 1.16–1.73), being older than 24 years (e.g., AOR = 9.57, 95% CI 6.61–13.86 for age 35–44 vs. 15–24 years) and use of condoms over the past 12 months (AOR = 1.56, 95% CI 1.28–1.91). Having tertiary education was associated with reduced odds of HIV infection (AOR = 0.41, 95% CI 0.27–0.66) (Table 2). 

### 3.2. Distribution of HIV Cases

**Figure 1:**
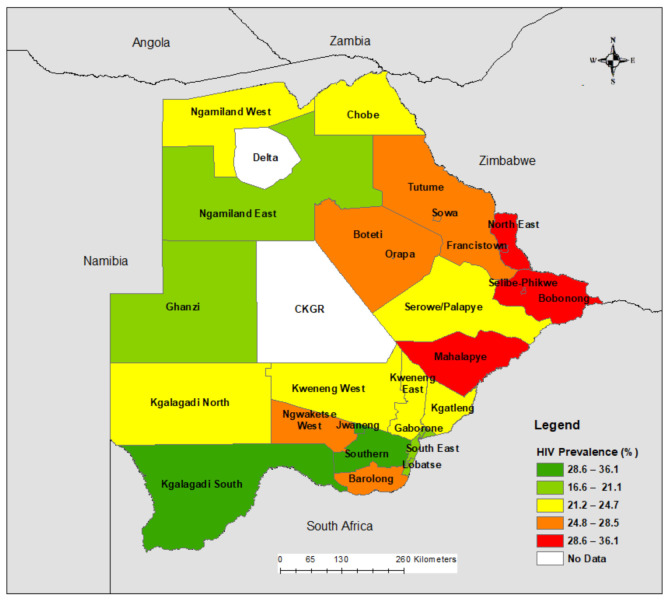
Crude HIV prevalence in adults by district in Botswana.

The crude estimation of HIV prevalence ranged from 15.5% in Jwaneng district to 36.1% in Mahalapye district (Figure 1). North East, Selibe-Phikwe and Bobonong districts also had high HIV prevalence—32.6%, 32.4% and 32.3% respectively (Figure 1). 

### 3.3. Identification of Spatial Clusters in Botswana

**Figure 2:**
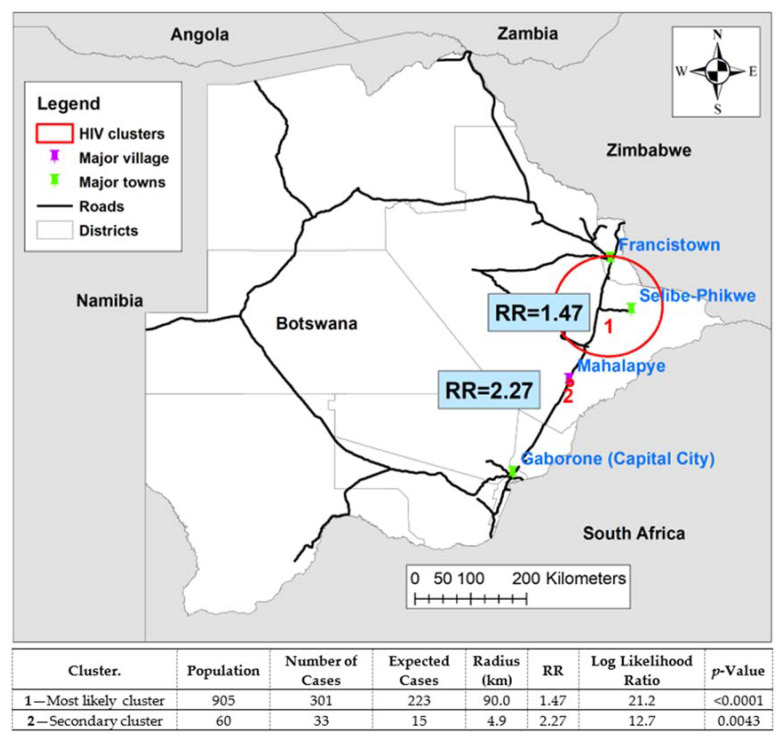
Clusters with high HIV prevalence in Botswana and summary statistics of significant clusters from SaTScan using a Bernoulli probability model.

The most likely cluster, cluster 1, was observed in the north-eastern region of Botwana and covers mostly Selibe-Phikwe and Francistown census districts as well as parts of Serowe/Palapye, North East and Central Bobonong census districts with a radius of 90.0 km. An RR of 1.47 (*p* < 0.0001) was estimated, implying that those in the 519 locations in this cluster had 47% increased risk of HIV compared with those outside the cluster. A secondary cluster, cluster 2, with a radius of 4.89 km, was located in Central Mahalapye census district in the central part of Botswana. The estimated RR of 2.27 (*p* = 0.004) implies that the population residing in the 34 locations within this cluster were 2.27 times more likely to be infected with HIV than those outside the cluster (Figure 2). A comparative analysis of the characteristics of clusters and non-clusters showed that more participants inside the cluster resided in rural areas (48% vs. 32%) and were unemployed (52% vs. 44%). The cluster communities also had fewer participants with tertiary education (10% vs. 20%) (Table 3).

## 4. Discussion

The study showed that the populations at greater risk of HIV infection were female, those aged older than 24 years and those who consistently used condoms in the past 12 months, while those with a higher education qualification were at lower odds of getting HIV. The scan statistics identified two HIV clusters independent of district boundaries, illustrating the presence of areas with high levels of HIV transmission in north-eastern Botswana census districts and Central Mahalapye census district.

Our results confirm that the HIV epidemic in Botswana is not ubiquitous but instead is characterised by geographically distinct clusters with disproportionately high numbers of people living with HIV. Cluster 1 covers a large area which encompasses a mining town, Selibe-Phikwe, the country’s second city Francistown and mostly villages in between and around these urban areas. Higher HIV prevalence in mining towns compared to other towns has been reported in southern African countries [15]. In their study, Carrel et al. observed a slowly shifting pattern of HIV prevalence increase in rural areas when compared with major cities in the Democratic Republic of the Congo between 2007 and 2013 [25]. They also found out that HIV prevalence increased rapidly in urban areas during the early stages of the HIV epidemic, before diffusing to rural areas. Further, proximity to the urban area was a determining factor for HIV spread into neighbouring areas [25], and this seems to be the case in the north-eastern parts of Botswana. Additionally, cluster 1 is located in close proximity to the country’s border with Zimbabwe, a country which also reports a high HIV prevalence rate, estimated at 16.7% in the 15–49 years age group [26]. The ongoing economic difficulties in Zimbabwe has prompted an influx of undocumented immigrants into the northern-eastern parts of Botswana and migration has been identified as a strong single predictor of high HIV prevalence and risk [27,28].

Cluster 2, on the other hand, is smaller, and found in a few suburbs in Mahalapye. Mahalapye is situated along a major national road, which also passes through cluster 1. Being half-way between the country’s capital city and Francistown (second city), Mahalapye is commonly used as a truck stop over by long-distance truck drivers. Studies have shown that long-distance truck drivers and people living in the vicinity of truck stops along major national roads are more at risk of contracting HIV when compared with the general population [29]. Our finding of identifying clusters along the national road is consistent with a study conducted by Tanser et al. in South Africa which identified clusters and high incidence of HIV along a national road [30].

Controlling for this spatial variability, our study reveals that women were at a higher risk of HIV infection in Botswana when compared with men. The findings are consistent with other studies conducted in SSA where HIV prevalence was found to be higher in females than males [2,26]. However, this is in contrast to findings in Western settings such as Australia, where women accounted for only 22% of new infections in 2017 [31]. In addition to biological factors, a wide range of economic and socio-cultural factors are responsible for increasing the vulnerability of African women to HIV infection [32]. To validate this, a study that examined sexual practices of ethnic groups in Botswana concluded that deeply rooted sexual norms and practices render women vulnerable to HIV infection, as these women do not have a say in pertinent sexual decisions such as the use of a condom [33]. Another factor that might contribute to males being less vulnerable in Botswana is SMC—an ‘add-on’ HIV prevention strategy that benefits men in Botswana. Studies from randomised control trials in Kenya, Uganda and South Africa have demonstrated that SMC can reduce the risk of acquiring HIV infection by 60% [34]. In addition to scaling up preventive strategies that protect women from HIV infection such as pre-exposure prophylaxis (PrEP), there is a need for the country to improve women’s economic and educational status [35]. Education has been found to increase opportunities for employment and gender equality for women which heightens their prospect of protection against HIV infection [32].

Our findings also show a significant association between HIV risk and increase in age. Those aged 15–24 years had a lower risk of HIV compared with older age groups. The current finding is in agreement with the results of a study that analysed BIAS III data [15]. The 25 years and above age group consists of those in their prime reproductive age, which could be associated with greater exposure to increased risk of HIV [36]. The reduced HIV risk in the 15–24 years age group could be the result of intensive age-focused strategies targeting this group, which was previously identified as having the highest transmission rates in Botswana [37]. ‘Life-skills’ education, a sexual risk reduction intervention, has been implemented as part of the curriculum in schools to increase adolescents’ HIV prevention skills and knowledge [38]. The continuing low HIV prevalence in this age group indicates the effectiveness of age-appropriate interventions, suggesting that such interventions should be given to older age groups to reduce the disease burden. 

The observed protective association between tertiary education and risk of HIV infection suggests that increased knowledge and economic independence are vital in HIV prevention. A study conducted in Botswana found that even though HIV awareness was high in the population, HIV knowledge was quite low. HIV knowledge is crucial in adopting and maintaining behaviours related to reduced risk of HIV [14]. Another study conducted in Zambia found a significant association between educational attainment and testing for HIV, such that testing was high among educated women living in urban areas, with wealth index further strengthening the association [39]. These findings indicate that there is a need for HIV interventions targeting those with lower education attainment to reduce their risk of HIV infection. Letshwenyo-Maruatona et al. recommended inclusion of innovative technology such as Facebook and YouTube to disseminate HIV/AIDS information and improve HIV knowledge in the general population [40]. However, as a long-term measure, the Botswana government should review its policies and introduce structural interventions to improve educational attainment [6]. 

An unexpected observation was that of increased risk of HIV infection among participants who used condoms consistently over the previous 12 months. Consistent with these findings are those reported in the settings of Malawi and South Africa [36,41]. However, the high HIV prevalence in those who reported to be consistently using condoms could be linked to social desirability bias, such that participants felt compelled to state that they use condoms when in fact they did not [42]. One more possible explanation could be bias due to the use of condoms by those already infected in a bid to protect their partners [5]. Thus, we do not interpret these findings of our study as suggesting that not using a condom is truly associated with lower HIV risk, but that the link is attributable to bias or other factors not captured in the model. Previous studies have found that condom use has been declining over the years in Botswana and one of the reasons cited is misconceptions that SMC gives protection against HIV. In a study conducted in Botswana, Namibia and Swaziland, Andersson and Crockcroft discovered that one in six of circumcised men believed that it was appropriate for a circumcised man to assume sex without a condom [43]. Government needs to re-scale-up programs that encourage use of condom in conjunction with educational HIV prevention interventions that would dispel this misconception.

The results of our study justify the inclusion of a geo-spatial method for in-depth analyses of HIV epidemics and associated covariates. Knowing which population and where it is located is imperative in the design of tailored and effective interventions as well as estimating region-specific needs for ART and other related services. Our results therefore suggest that policy makers should pay more attention to the north-eastern and central census districts particularly rural villages in close proximity to urban areas (cities and towns) and national roads by upscaling age-specific HIV education and testing to initiate early ART. Additionally, there is a need to address the influx of undocumented migrants in the country especially in the northern parts of Botswana. With regards to HIV-associated factors, more resources and appropriate interventions need to be directed towards sub-populations with a high disease burden such as women, those aged 25 years and above and people with low educational attainment. 

### Strengths and Limitations

There are a number of limitations to this study. First, the data are quite old (2013), so trends of HIV might have changed. However, this is the latest national representative survey and the findings from this study can be useful for program management. Secondly, the study being a cross-sectional design limits the ability to draw conclusions concerning temporality and causality of the observed association between the HIV infection and predictor variables. Further, as the survey data on sexual behaviour were based on self-reporting, they are prone to recall and reporting bias as well as social desirability bias. Thirdly, unmeasured risk factors not adjusted for during data analysis might have contributed to the association between HIV prevalence and its association covariates. The fourth limitation to be considered when interpreting the study results is the circular nature of the SaTScan window, which at times does not work well where neighbourhood-level geographic barriers could not create non-circular interaction patterns. Even with these limitations, the use of the geostatistical approach, in addition to the statistical analysis using a nationally representative sample (BIAS IV), are the major strengths of this study. Importantly, we have identified high-risk areas of HIV in Botswana, which were not reported in earlier studies. This finding will be useful for focused targeting of prevention measures for control of HIV in Botswana.

## 5. Conclusions

The study findings affirm that the HIV epidemic in Botswana is heterogenous across the country. The associated covariates of HIV prevalence include women, older population and low educational attainment. These results have important implications for planning and prevention programs in Botswana. The government need to move from a homogenous approach in resource allocation and intervention strategy to spatially focused and high-risk groups to curb the spread of HIV in Botswana.

## Figures and Tables

**Table 1:** Characteristics of study participants.

Variable	n (Weight %)	Proportion of HIV+	*p*-Value ^a^
Gender			
Female	2653 (56.4)	15.6	<0.001
Male	2055 (43.6)	9.5	
Age group			<0.001
15–24	1534 (32.6)	2.5	
25–34	1275 (27.1)	7.7	
35–44	902 (19.1)	8.4	
45–54	602 (12.8)	4.7	
55+	395 (8.4)	1.8	
Place of residence			0.6065
Urban	3040 (64.6)	16.0	
Rural	1668 (35.4)	9.1	
Education level			<0.001
None	465 (9.9)	2.8	
Primary	950 (20.2)	7.5	
Secondary	2440 (51.8)	12.2	
Tertiary	853 (18.1)	2.6	
Marital status			<0.001
Married	2066 (43.9)	13.4	
Never married	2642 (56.1)	11.7	
Religion			0.747
Christianity	3966 (84.2)	21.1	
Other	742(15.8)	4.0	
Employed			
Yes	2525 (53.6)	15.7	<0.001
No	2183 (46.6)	9.4	
Alcohol use			
Yes	1643 (34.9)	8.6	0.584
No	3065 (65.1)	16.5	
Condom use			
Yes	1991(42.3)	12.9	<0.001
No	2717(57.7)	12.3	

Note: ^a^ *p*-value for bivariate association between outcome and covariates (chi-square/two-sample *t*-test).

**Table 2:** Univariate and multivariable logistic regression models of risk factors associated with human immunodeficiency virus (HIV) infection.

Variables	Univariate Regression			Multivariable Regression		
	OR ^†^	CI *	*p*-Value	OR ^‡^	CI	*p*-Value
Gender						
Male	Ref			Ref		
Female	1.38	1.15–1.65	0.001	1.42	1.16–1.73	0.001
Age group						
15–24	Ref			Ref		
25–34	4.76	3.51–6.45	<0.001	5.04	3.60–7.05	<0.001
35–44	9.29	6.79–12.7	<0.001	9.57	6.61–13.9	<0.001
45–54	6.97	4.96–9.81	<0.001	7.07	4.59–10.9	<0.001
55+	3.36	2.25–5.03	<0.001	3.58	2.20–5.83	<0.001
Marital status						
Never Married	Ref			Ref		
Married	0.60	0.50–0.71	<0.001	1.02	0.83–1.27	0.831
Education						
None	Ref			Ref		
Primary	1.52	1.10–2.11	0.011	1.34	0.95–1.89	0.094
Secondary	0.80	0.59–1.09	0.161	1.11	0.77–1.60	0.56
Tertiary	0.43	0.29–0.65	<0.001	0.42	0.27–0.66	<0.001
Place of Residence						
Rural	Ref			Ref		
Urban	0.95	0.80–1.4	0.584	-	-	-
Religion						
Christian	Ref			Ref		
Other	1.04	0.81–1.33	0.749	-	-	-
Employment						
No	Ref			Ref		
Yes	0.62	0.52–0.74	<0.001	1.05	0.85–1.30	0.664
Alcohol use						
No	Ref			Ref		
Yes	0.95	0.79–1.15	0.584	-	-	-
Condom use						
No	Ref			Ref		
Yes	1.62	1.36–1.94	<0.001	1.56	1.28–1.91	<0.001

Note: ^†^ Odds ratio; ^‡^ Adjusted odds ratio; * Confidence interval.

**Table 3:** Comparison of socio-demographic characteristics between participants within and outside the clusters.

Variable	Inside the Clusters (%)	Outside the Clusters (%)	*p*-Value
HIV positive	30	24	0.012
Gender			0.222
Female	54	57	
Male	46	43	
Age group			0.559
15–24	30	33	
25–34	27	27	
35–44	19	19	
45–54	15	13	
55+	9	8	
Place of residence			<0.001
Urban	52	68	
Rural	48	32	
Education level			0.001
None	13	9	
Primary	26	19	
Secondary	51	52	
Tertiary	10	20	
Marital status			0.885
Married	44	44	
Never married	56	56	
Religion			<0.001
Christianity	76	86	
Other	24	13	
Employed			0.002
Yes	48	55	
No	52	44	
Alcohol use			0.779
Yes	35	35	
No	65	65	
Condom use			<0.312
Yes	44	42	
No	55	58	

## Data Availability

Data supporting the results can be obtained upon request from Ministry of Health and Wellness Botswana.
